# Surgical site infections after distal radius fracture surgery: a nation-wide cohort study of 31,807 adult patients

**DOI:** 10.1186/s12891-020-03822-0

**Published:** 2020-12-18

**Authors:** Johanna Rundgren, Anders Enocson, Hans Järnbert-Pettersson, Cecilia Mellstrand Navarro

**Affiliations:** 1 Department of Clinical Science and Education, Södersjukhuset, Karolinska Institutet, SE-118 83 Stockholm, Sweden; 2 Department of Molecular Medicine and Surgery, Karolinska Institutet, SE-171 76 Stockholm, Sweden

**Keywords:** Distal radius fracture, Surgical site infection, Postoperative infection, Infection after fracture fixation, Plate fixation, Percutaneous pinning, Pin fixation, External fixation, Complications, Antibiotics

## Abstract

**Background:**

Surgical site infections (SSI) after distal radius fracture (DRF) surgery have not previously been studied as the primary outcome in a large population with comparative data for different surgical methods. The aims of this study were 1) to compare SSI rates between plate fixation, percutaneous pinning and external fixation, and 2) to study factors associated with SSI.

**Methods:**

We performed a nation-wide cohort study linking data from the Swedish national patient register (NPR) with the Swedish prescribed drug register (SPDR). We included all patients ≥18 years with a registration of a surgically treated DRF in the NPR between 2006 and 2013. The primary outcome was a registration in the SPDR of a dispensed prescription of peroral Flucloxacillin and/or Clindamycin within the first 8 weeks following surgery, which was used as a proxy for an SSI. The SSI rates for the three main surgical methods were calculated. Logistic regression was used to study the association between surgical method and the primary outcome, adjusted for potential confounders including age, sex, fracture type (closed/open), and a dispensed prescription of Flucloxacillin and/or Clindamycin 0–8 weeks prior to DRF surgery. A classification tree analysis was performed to study which factors were associated with SSI.

**Results:**

A total of 31,807 patients with a surgically treated DRF were included. The proportion of patients with an SSI was 5% (*n* = 1110/21,348) among patients treated with plate fixation, 12% (*n* = 754/6198) among patients treated with percutaneous pinning, and 28% (*n* = 1180/4261) among patients treated with external fixation. After adjustment for potential confounders, the surgical method most strongly associated with SSI was external fixation (aOR 6.9 (95% CI 6.2–7.5, *p* < 0.001)), followed by percutaneous pinning (aOR 2.7 (95% CI 2.4–3.0, *p* < 0.001)) (reference: plate fixation). The classification tree analysis showed that surgical method, fracture type (closed/open), age and sex were factors associated with SSI.

**Conclusions:**

The SSI rate was highest after external fixation and lowest after plate fixation. The results may be useful for estimation of SSI burdens after DRF surgery on a population basis. For the physician, they may be useful for  estimating the likelihood of SSI in individual patients.

## Background

The main surgical methods for distal radius fractures (DRFs) include open reduction with internal fixation (ORIF) using a volar plate (plate); closed reduction with percutaneous pinning (pins); and closed reduction with external fixation (EF). While a majority of all DRFs can be managed non-surgically, about 20% are treated surgically [1, 2]. Recent studies have reported that the proportion of surgically managed fractures is increasing, and that the preferred surgical method has shifted from percutaneous techniques to ORIF over the last two decades [1–7].

Many studies have compared surgical methods for displaced DRFs with regard to functional, radiographic and patient-reported outcome measures in a variety of settings and patient groups. While long-term functional and patient-reported outcomes have been shown to be similar between the most frequently used surgical methods [8–14], the spectrum and frequency of associated complications vary considerably [15–17]. It is therefore paramount that physicians treating patients with a displaced DRF are familiar with the complications associated with each treatment method in order to achieve the most favorable outcome.

Surgical site infection (SSI) is a well-known and dreaded complication after orthopedic surgery [18]. Several variables may influence the development of an infection after fracture fixation, including fracture-related factors (closed/open fracture type, complexity of the fracture, status of the surrounding soft tissues), patient-related factors (age, comorbidities, medication, nutritional status, tobacco use), and procedure-related factors (correct use of perioperative antibiotic prophylaxis, surgical technique, sterility, hygiene in the operating room) [18–20]. Not only do orthopedic SSIs cause an additional economic burden to the health-care system [21], but they may also result in delayed healing, functional loss and protracted recovery periods for individual patients [18, 19].

There is great heterogeneity in the literature with regard to the definition and standard criteria of SSI [18–20]. A frequently cited definition has been provided by the American Center for Disease Control and Prevention (CDC), subdividing SSIs based on the depth of tissue involvement at diagnosis into: superficial incisional, deep incisional and organ space [22]. According to the CDC definition, an SSI must occur within 30 days of surgery, unless a foreign material has been implanted, in which case the time frame is 1 year [22]. A widely used classification scheme for SSI, which has proved clinically relevant in the treatment of infections after fracture fixation, is based on the time of onset after surgery: early (< 2 weeks), delayed (2–10 weeks) and late (> 10 weeks) [18, 23]. Early onset SSIs after fracture fixation are primarily caused by the high-virulence pathogen *Staphylococcus aureus*, often presenting with classic symptoms of infection (i.e. pain, swelling, redness, secretion and/or pus), while late onset SSIs are primarily caused by low-virulent bacteria capable of producing biofilm, such as *Staphylococcus epidermidis*, often presenting with more subtle symtoms [18, 20].

With regard to DRF surgery, most SSIs are superficial and occur early on, e.g. pin site infections in patients treated with percutaneous pinning or external fixation, and are manageable with peroral antibiotics [11, 16]. However, if not treated at an early stage, they may develop into deep SSIs with resulting osteomyelitis and non-union, requiring in-patient care with intravenous antibiotics and repeated surgical site debridement [16].

The available data on the occurrence of SSI after DRF surgery consist of either retrospective case series without a comparator, or prospective clinical trials with SSI studied as a secondary outcome. To our knowledge, there is no prior publication with SSI rates after DRF surgery as the primary outcome in a large population with comparative data for different surgical methods.

The aims of this study were 1) to compare SSI rates after DRF surgery between the three most established surgical methods, i.e. plate fixation, percutaneous pinning and external fixation, and 2) to study factors associated with SSI.

## Methods

### Study setting and data sources

The unique personal identity number given to all Swedish residents at birth or on a residence permit enables linkage between population-basedhealth-care registers, thus providing comprehensive combined data for epidemiological studies.

The Swedish national patient register (NPR) is maintained by the Swedish national board of health and welfare, and provides patient data, geographical data, administrative in- and out-patient data, as well as medical data including main and secondary diagnoses and surgical procedure codes [24]. National coverage of in-patient care was achieved in 1987. Reporting surgical interventions became mandatory for health-care providers in 1993. Day-care surgery was added to the register in 1997, followed by all other specialized (hospital-based) out-patient care in 2001. Since 2001 the NPR includes data from both public and private caregivers. Patients’ diagnoses are registered using the Swedish version of the international statistical classification of diseases and related health problems 10th revision (ICD-10-SE) code system. Surgical procedures are recorded in accordance with the Nordic-medico-statistical committee (NOMESCO) classification of surgical procedures, Swedish version (NCSP-S) [25]. The quality of data for in-patient care is considered good and the drop-out rate for the main variables has been approximately 1% since 1987. For specialized out-patient care, the quality and drop-out rate for the main variables have improved greatly since 2001, with a decrease in drop-out rate from 25 to 30% to approximately 3% [26].

The Swedish prescribed drug register (SPDR) is maintained by the Swedish national board of health and welfare [27]. It provides nation-wide detailed data on all prescribed drugs dispensed at Swedish pharmacies, including the anatomical therapeutic chemical (ATC) classification code, date of prescription and date of dispensation. Since July 2005 the SPDR includes personal identity numbers, thus allowing linkage to other health-care registers.

Sweden has a restrictive national policy regarding prescription and use of antibiotics [28]. Antibiotic prophylaxis in orthopedic surgery is usually administered as a perioperative intravenous bolus 1 to 3 doses regimen [29]. Peroral antibiotic prophylaxis continuing after these doses is not recommended [29]. Within the context of SSI after DRF surgery, given that most SSIs are superficial and occur early on [11, 16], the clinical praxis in the setting under study is peroral treatment primarily aimed at *Staphylococcus aureus* with the isoxazolylpenicillin Flucloxacillin or, in case of penicillin allergy, with the lincosamide Clindamycin, both of which are available only after prescription.

### Study design and period

This was a nation-wide observational cohort study linking prospectively registered data from two population-based Swedish health-care registers, the NPR and the SPDR. The study period was November 1st 2006 to October 31st 2013.

### Inclusion and exclusion criteria of study population

The original NPR data file contained unselected data on all forearm fractures in Sweden including the years 2001–2013, i.e. all registrations beginning with the ICD-10-SE code S52, for both in- and out-patient care. The original SPDR file included data for the time period 2005–2013.

#### Inclusion criteria

The present study included all patients aged 18 years or older with a surgically treated DRF registered in the NPR between November 1st 2006 and October 31st 2013.

A DRF was defined as a registration of the ICD-10-SE codes S525 or S526, as a main and/or secondary diagnosis. The surgical methods were defined as a registration of any of the following NCSP-S codes: NCJ29, NDJ29 = external fixation; NCJ49, NDJ49 = percutaneous pinning; NCJ69, NDJ69 = plate fixation.

The index date (date of surgery) was defined as the first registration of a relevant surgical procedure code (NCJ29, NDJ29, NCJ49, NDJ49, NCJ69 or NDJ69) with a concomitant relevant DRF code (S525 or S526), occurring within 28 days of the “first” registration of a DRF code in the NPR. The 28-day time limit between diagnosis and surgery was chosen to represent a clinically relevant interval for primary fracture surgery. The “first” DRF registration was defined as the occurrence of a DRF code preceded by a period of at least 18 months during which no DRF code occurred in the NPR. If a patient had several index dates during the study period, only the first surgical treatment (first index date) was included. Concomitant bilateral fractures were analyzed as one unilateral fracture. Thus, each unique patient was only included once and the number of DRFs in our data equals the number of patients.

#### Exclusion criteria

The early years in the original NPR data file were excluded because linkage to the SPDR with personal identity numbers was only possible from July 2005, and because of the potentially high drop-out rate of out-patient data during that period. Furthermore, the study period ended 8 weeks before the end of 2013 to allow for screening of the primary outcome in the SPDR for all included patients.

We excluded all forearm fractures other than DRFs, as well as all non-surgically treated DRFs. Furthermore, we excluded all index dates where surgery was not performed with any of the main established surgical methods, including the following NCSP-S codes: NCJ89/NDJ89 = combinations, and NCJ59/NDJ59/NCJ99/NDJ99 = other methods.

In order to rid our data from secondary surgical treatments due to sequele from a previous DRF treatment, we excluded all index dates occurring more than 28 days after the first DRF code, as well as if the first DRF code was not preceded by a period of ≥18 months without a DRF code registration. As a result, we screened the NPR file from May 1st 2005 to October 31st 2006.

**Fig. 1:**
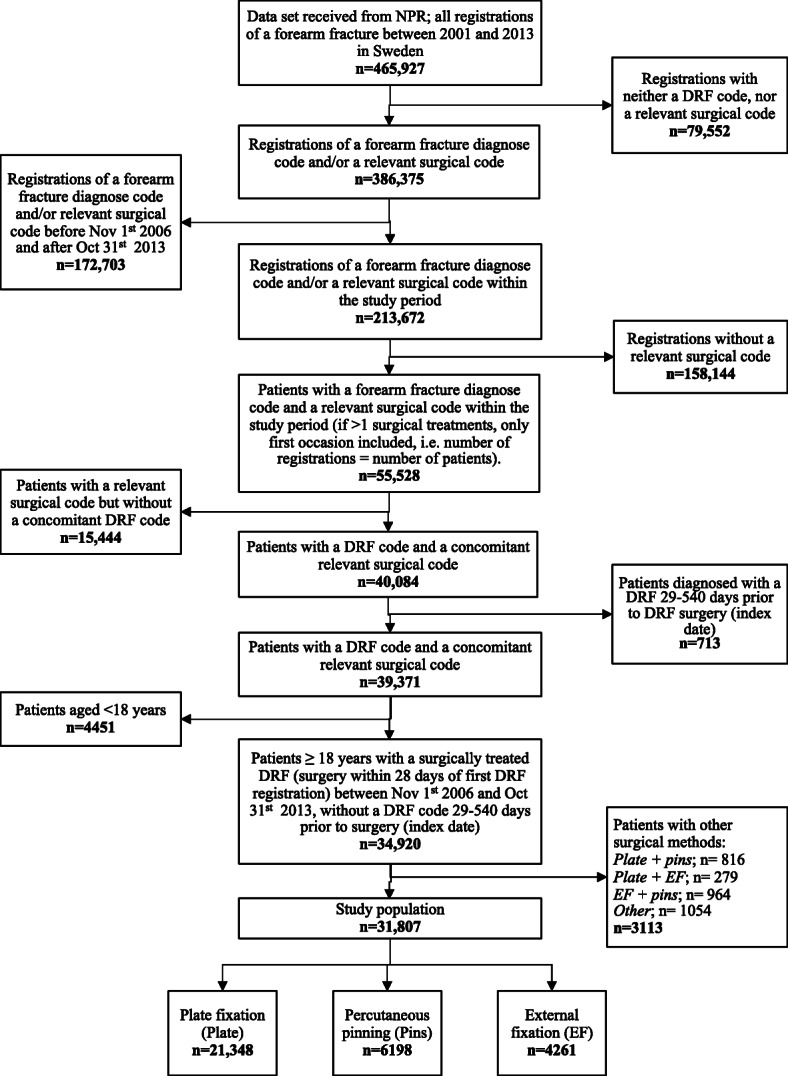
Flowchart illustrating the step-by-step selection of the study population consisting of all patients aged 18 years or older with a distal radius fracture (DRF) treated with either plate fixation, percutaneous pinning or external fixation, and registered in the NPR between November 1st 2006 and October 31st 2013

Figure 1 visualizes the step-by-step process of selecting the study population.

### Exposures

Included patients were allocated to either of three exposure groups based on surgical method: plate fixation, percutaneous pinning or external fixation.

### Primary outcome

The primary outcome was a registration in the SPDR of a dispensed prescription of peroral Flucloxacillin and/or Clindamycin within the first 8 weeks following DRF surgery (yes/no), which was used as a proxy for an SSI. We used the ATC codes for Flucloxacillin (J01CF05) and Clindamycin (J01FF01) to identify the drugs in the SPDR. The primary outcome was defined as yes if the patient had an occurrence of the ATC codes J01CF05 and/or J01FF01 in the SPDR within the first 8 weeks following the index date. We defined the timing of the primary outcome as the date of prescription, as opposed to the date of dispensation.

### Potential confounders

Data on patient age and sex were extracted from the NPR. Study patients were categorized into three age groups: 18–49 years, 50–74 years and ≥ 75 years.

The fifth position in the ICD-10-SE code system indicates fracture type, i.e. a closed (0) or open (1) fracture. These data were also extracted. The fracture type was classified as closed if data in the fifth position were missing.

To assess the proportion of individuals in the study population already under treatment with Flucloxacillin and/or Clindamycin due to other reasons at the time of their DRF, we analyzed the occurrence (yes/no) of a dispensed prescription of Flucloxacillin and/or Clindamycin in the SPDR within 8 weeks prior to the index date, using ATC codes.

Table 1 contains an overview of the definitions for all variables included in the study.

**Table 1:** Definitions of variables in a nation-wide cohort study of surgical site infection rates and associated factors in 31,807 adult patients undergoing surgery of a distal radius fracture in Sweden between November 1st 2006 and October 31st 2013

Variable	Data source	Definition
**Inclusion criteria**	NPR	Patients ≥18 years with a surgically treated DRF registered in the NPR between Nov 1st 2006 and Oct 31st 2013
DRF	NPR	ICD-10-SE codes S525 and/or S526 as primary and/or secondary diagnosis
Surgical methods	NPR	NCSP-S codes: NCJ29, NDJ29 = external fixation. NCJ49, NDJ49 = percutaneous pinning. NCJ69, NDJ69 = plate fixation
Index date	NPR	First concomitant registration of a code for a relevant surgical procedure (NCJ29, NDJ29, NCJ49, NDJ49, NCJ69 or NDJ69) *and* a DRF code (S525 or S526), occurring within 28 days from the first DRF registration (S525, S526). If several index dates, only first index date included
First DRF registration	NPR	First registration of a DRF code (S525 or S526) preceded by a period ≥18 months without any registration of a DRF code. NPR was screened from May 1st 2005 with regard to the 18-month period
**Exclusion criteria**		
Time periods	NPR	Index date occurring between Jan 1st 2001 - Oct 31st 2006 and Nov 1st 2013 - Dec 31st 2013
Forearm fractures other than DRFs	NPR	ICD-SE-10 codes beginning with S52, other than S525 and S526
Non-surgically treated DRFs		Registrations of a DRF code (S525, S526) without a concomitant surgical procedure code
DRFs treated with other surgical methods	NPR	NCSP-S codes: NCJ89, NDJ89 = combinations. NCJ59, NDJ59, NCJ99, NDJ99 = other methods
Non-adults	NPR	Age < 18 years
**Primary outcome**	SPDR	Yes/No. Yes = a registration of the ATC codes J01CF05 (Flucloxacillin) and/or J01FF01 (Clindamycin) in the SPDR within the first 8 weeks following the index date. The date of primary outcome = date of prescription
**Potential confounders**		Factors used to adjust the association between surgical method and the primary outcome
Age	NPR	At the time of index date. Categorized into 18–49 years, 50–74 years, and ≥ 75 years
Sex	NPR	Women/Men
Fracture type	NPR	Closed/open. Fifth position in ICD-SE-10 code. Closed = 0, Open = 1. If missing defined as closed (0)
Antibiotics treatment due to other reasons at the time of DRF surgery	SPDR	Yes/No. Yes = registration of the ATC codes J01CF05 (Flucloxacillin) and/or J01FF01 (Clindamycin) in the SPDR during the 8 weeks preceding the index date

*DRF* distal radius fracture, *NPR* Swedish national patient register, *SPDR* Swedish prescribed drug register, *NCSP-S* NOMESCO classification of surgical procedures, Swedish version, *ATC* anatomical therapeutic chemical classification code

### Statistical analysis

Data are presented as numbers and percentages for each surgical method. Logistic regression was used to study the association between the primary outcome and surgical method adjusted for possible confounders: age, sex, fracture type (closed/open) and a dispensed prescription of Flucloxacillin and/or Clindamycin 0–8 weeks prior to DRF surgery. Odds ratios (ORs) were presented for both the uni- and multivariable analyses, with 95% confidence intervals (CI) and corresponding *p*-values. Results were considered significant at *p* < 0.05 in 2-sided tests. In addition, we used classification tree analysis adjusted according to Bonferroni to study which factors were associated with the primary outcome. The variables included in the tree analysis were surgical method, fracture type (closed/open), age (18–74 and ≥ 75 years), sex, and a dispensed prescription of Flucloxacillin and/or Clindamycin 0–8 weeks prior to DRF surgery (yes/no). The limit on the depth of the classification tree was set to four node levels. The statistical software used for all analyses was IBM SPSS Statistics, version 23 and 25 for Windows.

## Results

A total of 31,807 patients 18 years or older with a surgically treated DRF registered in the NPR between November 1st 2006 and October 31st 2013 were identified. Of these, 21,348 were registered as plate fixation, 6198 as percutaneous pinning and 4261 as external fixation. Basic characteristics of the study population are presented in Table 2.

**Table 2:** Demographics of study population; 31,807 adult patients undergoing surgery of a distal radius fracture in Sweden between November 1st 2006 and October 31st 2013

Variables		Plate^**a**^ n (%)	Pins^**b**^ n (%)	EF^**c**^ n (%)	All n (%)
**Age (years)**	**18–49**	5016 (24%)	1197 (19%)	558 (13%)	6771 (21%)
	**50–74**	13,088 (61%)	3581 (58%)	2437 (57%)	19,106 (60%)
	**≥75**	3244 (15%)	1420 (23%)	1266 (30%)	5930 (19%)
**Sex**	**Women**	16,517 (77%)	5124 (83%)	3518 (83%)	25,159 (79%)
	**Men**	4831 (23%)	1074 (17%)	743 (17%)	6648 (21%)
**Fracture type**	**Closed**	20,969 (98%)	6131 (99%)	4072 (96%)	31,172 (98%)
	**Open**	379 (2%)	67 (1%)	189 (4%)	635 (2%)
**Antibiotics**^**d**^ **0–8 weeks prior to DRF surgery**	**No**	20,947 (98%)	6110 (99%)	4169 (98%)	31,226 (98%)
	**Yes**	401 (2%)	88 (1%)	92 (2%)	581 (2%)

^a^ Plate fixation. ^b^ Percutaneous pinning. ^c^ External fixation. ^d^ A dispensed prescription of Flucloxacillin and/or Clindamycin. *n* numbers, *DRF* distal radius fracture

The overall proportion of patients with a dispensed prescription of peroral Flucloxacillin and/or Clindamycin within the first 8 weeks following DRF surgery, i.e. the SSI rate, was 10%. For the three main surgical methods respectively, the SSI rate was 5% after plate fixation, 12% after percutaneous pinning and 28% after external fixation. Numbers are presented in Table 3.

**Table 3:** Factors associated with a dispensed prescription of Flucloxacillin and/or Clindamycin within 8 weeks following surgery of a distal radius fracture between November 1st 2006 and October 31st 2013 in 31,807 adult Swedish patients. A logistic regression model was performed and results are presented as crude measures and odds ratios in uni- and multivariable analyses

Variable		Crude measures		Univariable		Multivariable *	
		Total n	Antibiotics^**d**^ 0–8 weeks after DRF surgery n (%)	OR	95% CI, ***p***-value	aOR	95% CI, ***p***-value
**Surgical method**	**Plate**^**a**^	21,348	1110 (5%)	Ref.		Ref.	
	**Pins**^**b**^	6198	754 (12%)	2.5	2.3–2.8, < 0.001	2.7	2.4–3.0, < 0.001
	**EF**^**c**^	4261	1180 (28%)	7.0	6.4–7.6, < 0.001	6.9	6.2–7.5, < 0.001
**Age (years)**	**18–49**	6771	564 (8%)	Ref.		Ref.	
	**50–74**	19,106	1640 (9%)	1.0	0.9–1.1, 0.520	1.1	1.0–1.3, < 0.038
	**≥75**	5930	840 (14%)	1.8	1.6–2.0, < 0.001	1.6	1.4–1.8, < 0.001
**Sex**	**Women**	25,159	2183 (9%)	Ref.		Ref.	
	**Men**	6648	861 (13%)	1.6	1.4–1.7, < 0.001	2.0	1.8–2.2, < 0.001
**Fracture type**	**Closed**	31,172	2776 (9%)	Ref.		Ref.	
	**Open**	635	268 (42%)	7.5	6.4–8.8, < 0.001	6.4	5.3–7.6, < 0.001
**Antibiotics**^**d**^ **0–8 weeks prior to DRF surgery**	**No**	31,226	2940 (9%)	Ref.		Ref.	
	**Yes**	581	104 (18%)	2.1	1.7–2.6, < 0.001	1.6	1.3–2.1, < 0.001

*Logistic regression model adjusted for surgical method, age, sex, fracture type (closed/open), and a dispensed prescription of Flucloxacillin and/or Clindamycin 0–8 weeks prior to DRF surgery. ^a^ Plate fixation. ^b^ Percutaneous pinning. ^c^ External fixation. ^d^ A dispensed prescription of Flucloxacillin and/or Clindamycin. *OR* odds ratio, *aOR* adjusted odds ratio, *CI* confidence interval, *n* numbers, *Ref* reference. *DRF* distal radius fracture

Factors associated with the primary outcome (prescription of antibiotics as a proxy for SSI) are presented in Table 3. Prescription of antibiotics was strongly associated with external fixation and percutaneous pinning, with an adjusted OR (aOR) of 6.9 (CI 6.2–7.5, *p* < 0.001) for external fixation, and an  aOR of 2.7 (CI 2.4–3.0, *p* < 0.001) for percutaneous pinning, compared with plate fixation (reference). Open fracture type (aOR 6.4 (CI 5.3–7.6, *p* < 0.001)) and male sex (aOR 2.0 (CI 1.8–2.2, *p* < 0.001)) were also associated with  the primary outcome.

**Fig. 2:**
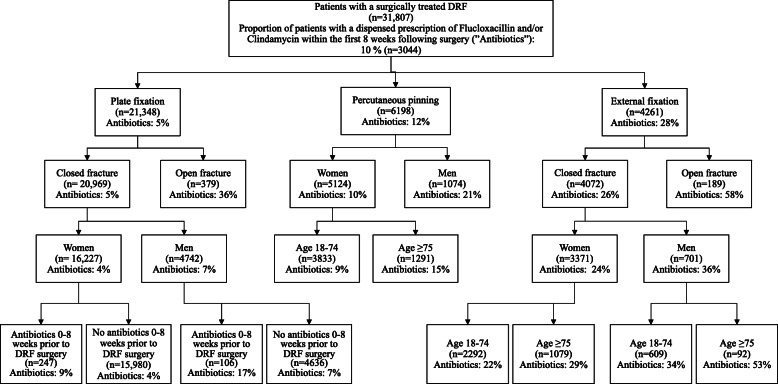
Classification tree showing the factors, which at each node level, had the strongest association with the primary outcome, i.e. a dispensed prescription of Flucloxacillin and/or Clindamycin (“Antibiotics”) within the first 8 weeks following surgical treatment of a distal radius fracture (DRF). The percentage in each box represents the proportion of patients with the primary outcome. *P*-values were < 0.001 at all nodes and adjusted according to Bonferroni

The classification tree analysis (Fig. 2) showed that surgical method, fracture type (closed/open), sex and age were factors associated with the prescription of antibiotics. The proportion of patients with prescription of antibiotics was 58% for patients undergoing external fixation of an open fracture, while it was 36% for plate fixated patients with an open fracture. Regardless of surgical method and node level, the proportion of men with a prescription of antibiotics was higher than for women, e.g. 21% among men compared to 10% among women undergoing percutaneous pinning, and 36% among men and 24% among women undergoing external fixation of a closed fracture.

## Discussion

This nation-wide cohort study showed that the rate of SSI after DRF surgery was highest among patients undergoing external fixation (28%), followed by percutaneous pinning (12%) and plate fixation (5%). In addition, the classification tree analysis showed that surgical method, fracture type (closed/open), sex and age were factors associated with the prescription of antibiotics (Flucloxacillin and/or Clindamycin). The highest proportion of antibiotics prescription was found among patients undergoing external fixation of an open fracture (58%), followed by externally fixated closed DRFs in men aged ≥75 years (53%).

### SSI rates – relation to previous research

The existing literature on DRF treatment and outcome is extensive and heterogenic. Several previous prospective randomized controlled trials (RCT) comparing the main surgical methods in the treatment of displaced DRFs with regard to patient-reported and functional outcomes have also presented data on the occurrence of SSIs. The reported SSI rates varied between 0 and 5.6% after plate fixation [8, 10–12, 17, 30], between 7.8 and 23% after percutaneous pinning [10, 11, 17], and between 5.3 and 26% after external fixation [8, 12, 17, 30]. The great variance in SSI rates for each surgical method between these studies may be explained by differences in inclusion and exclusion criteria (e.g. patient age, fracture classification and type), size of the study population, as well as the definition of SSI. Furthermore, these studies were designed to detect differences in clinical outcome, and SSI rates were presented only as secondary outcomes. Interestingly, it was the largest of these previous studies, a secondary analysis of 461 patients included in a multicenter pragmatic RCT and allocated to either volar plate fixation or percutaneous pinning, which reported SSI rates most in accordance with those in our study; 5.6% for plate fixation and 8.3% for percutaneous pinning [11].

A recent Cochrane systematic review of percutaneous pinning in the treatment of DRFs in adults [13], pooled data from 21 RCTs and 5 quasi-RCTs comparing either pinning with cast immobilization, different pinning techniques, or immobilization regimes for displaced or unstable DRFs in a total of 1946 patients, with regard to short-, medium- and long-termpatient-reported outcomes and complications. They reported an SSI rate of 7.7% (ranging from 0 to 15%) in the 285 patients treated with percutaneous pinning.

A meta-analysis comparing treatment outcomes and complication rates between volar plate fixation and percutaneous pinning in the treatment of dorsally displaced DRFs pooled data from seven RCTs with a total of 875 patients, and reported a rate of superficial SSI of 3.2% after volar plate fixation and 8.2% after percutaneous pinning, while the rate of deep SSI was 0.5% for both methods [31].

Another meta-analysis compared volar plate fixation to external fixation in the treatment of DRFs in adults by pooling data from nine RCTs with a total of 780 patients [32], and reported SSI rates of 0.5% after plating and 7.7% after external fixation. This was markedly below our findings of 5% and 28% respectively.

In contrast to our findings, a literature review from 2015 on the management of complications following DRF treatment reported a higher SSI rate after percutaneous pinning (33%) than after external fixation (21%) [16]. However, these numbers were based on two previously published studies, one of which prospectively compared the rates of pin tract infection between buried and percutaneous wires in 56 patients [33], and the other which retrospectively analyzed complications in 314 DRF patients treated with external fixation [34].

A previous retrospective study by van Leeuwen et al. analyzed the occurrence of pin site infection and associated factors in 1213 patients undergoing percutaneous pinning of fractures in the wrist and/or hand at one of three institutions, by reviewing medical charts [35]. They defined SSI either by early removal of pins, prescription of antibiotics for pin problems within 90 days, or surgery for infection related to the pin. The reported SSI rate was 7%, and a majority of infections were superficial and resolved with peroral antibiotics and/or pin removal.

### Associated factors – relation to previous research and further considerations

In our study, age was associated with prescription of antibiotics for patients treated with both percutaneous pinning and external fixation, with a higher proportion of antibiotic prescription among patients ≥75 years. In the previously mentioned study of factors associated with pin site infection by van Leeuwen et al. [35], high age was associated with infection in their bivariate analysis, however in their multivariable analysis, no individual factor (including age, smoking, fracture location, fracture mechanism or number of pins) was associated with increased or decreased odds for pin site infection. Furthermore, we found no association between age and antibiotic prescription for patients treated with plate fixation. This is supported by a recent retrospective study of age-related outcomes and complications in 105 patients (aged 17–80 years) treated with volar plate fixation [36], in which no significant difference in overall complication rate between patients aged younger or older than 55 years was found, and the rate of SSI was 3.4% for younger patients and 4.3% for older.

Our findings of an association between antibiotics prescription and fracture type (closed/open), with a higher proportion of antibiotics prescription among patients with an open fracture, were not surprising and in accordance with current knowledge and our clinical experience. They are explained by a well-established inherently increased risk of SSI due to contamination of the surgical site [18]. However, as open fractures may vary considerably in severity, we cannot exclude that some of these prescriptions were prophylactic.

Our study showed an association between male sex and prescription of antibiotics as indicated by an aOR of 2.0 in the multivariable logistic regression model, as well as a higher proportion of antibiotics prescription among men compared to women regardless of surgical method and node level in the classification tree analysis. To the best of our knowledge, SSI rates in relation to gender have not previously been studied. We speculate that our findings may be due to a higher percentage of high energetic trauma among men and thus an inherent higher risk of infection. Another possible explanation may be a tendency among health-care providers to treat men with prescribed antibiotics to a greater extent than women. Further studies are needed to investigate this.

### Aspects of study design and methods, strengths and limitations

The great number of included patients in this study warranted high precision. Further, the high coverage of the population-based registers (the NPR and the SPDR) reduced the risk of selection bias.

We chose to investigate SSI after DRF surgery by the use of a proxy because the coding of SSI after fracture fixation in the NPR was not considered consistent or reliable, most likely due to the previously reported lack of consensus on definition and classification in clinical practice [20]. Given the strongly regulated and well-monitored pharmaceutical system in Sweden [27], as well as the extensive multi-levelcross-sectoral national efforts to contain antibiotic resistance over the recent decades, in which the Swedish strategic program against antibiotic resistance (STRAMA) has played a central role [28], providing guidelines for antibiotic use, we believe that the use of a dispensed prescription of peroral Flucloxacillin and/or Clindamycin as a proxy was valid. Our motives included, firstly, that no over-the-counter antibiotics are permitted in Sweden. Secondly, antibiotic prophylaxis continuing after the perioperative intravenous doses, is not recommended in Sweden [29]. Thirdly, as most primary SSIs after DRF surgery are superficial and have an early onset [11, 16], the clinical praxis in the setting under study is peroral treatment primarily aimed at *Staphylococcus aureus,* with Flucloxacillin, or Clindamycin in case of penicillin allergy.

The time period after surgery during which the SPDR was screened for prescriptions of antibiotics was set to 8 weeks based on our clinical experience as well as previous research [18, 23, 37]. We are aware that by doing so, late onset SSIs occurring after 8 weeks were missed. However, based on our clinical experience we believe that late onset SSIs are rare in DRF surgery.

The study period encompassed 7 years, ending in 2013. We are aware that treatment trends have continued to change since 2013, with an increasing popularity of plate fixation over external fixation as the method of choice. However, we argue that by covering a time period during which external fixation was still one of the standard treatment methods for displaced non-complex DRFs, this study has provided comparative data for the main surgical methods, which is less susceptible to confounding by indication than more recent data would be.

A limitation of the NPR was its lack of detailed patient- and fracture-related data relevant to fracture treatment and complications (e.g. fracture side, fracture classification, tobacco use). Another potential limitation was the lack of validation of DRF codes in the NPR. Further, as patients with concomitant bilateral DRFs or a recurring DRF within the study period were only accounted for once, there was a risk of underestimating the number of fractures. Also, the NCSP-S code for plate fixation in the NPR is the same for volar, dorsal and multiple plating. While the standard approach for plate fixation of DRFs is volar, dorsal and multiple approaches are mainly used for a subset of complex DRFs. To not be able to separate these may have introduced bias. Lastly, the NCSP-S code for percutaneous pinning does not discriminate between pins buried under the skin or not, which also may have introduced bias. However, a recent Cochrane review of percutaneous pinning in the treatment of DRFs found very low-quality evidence that buried pins reduce the incidence of superficial SSIs [13].

The SPDR provides information on dispensed prescription drugs only. Thus, prescribed medications which are not dispensed are not registered in the SPDR. This may have caused an underestimation of the primary outcome in our study. Likewise, an overestimation of our primary outcome may have been caused by prophylactic prescription of antibiotics for postoperative swelling and pain without positive bacterial culture findings, as well as by prescription of Flucloxacillin and/or Clindamycin due to other infections unrelated to DRF surgery.

We did not extract the NCSP-S code for surgical debridement from the NPR file. Thus, in this register study we could not differ superficial SSIs manageable with only peroral antibiotics from deep infections requiring in-patient care, intravenous antibiotics and surgical debridement. However, deep SSIs which require surgical debridement and intravenous antibiotics are rare after DRF surgery [38]. Furthermore, these patients are most likely prescribed peroral antibiotics at discharge and are thus included in our study population.

## Conclusion

The SSI rate was highest after external fixation and lowest after plate fixation. The results may be useful for estimation of SSI burdens after DRF surgery on a population basis. For the physician, they may be useful for estimating the likelihood of SSI in individual patients.

## Data Availability

The dataset used during the current study is available from the corresponding author on reasonable request.

## Abbreviations

ATC: Anatomical Therapeutic Chemical classification code
CDC: American Centers for Disease Control and Prevention
CI: Confidence Interval
DRF: Distal Radius Fracture
EF: External Fixation
ICD-10-SE: International Statistical Classification of Diseases and related health problems 10th revision, Swedish version
NCSP-S: NOMESCO classification of surgical procedures, Swedish version
NOMESCO: Nordic-medico-statistical committee
NPR: Swedish National Patient Register
OR, aOR: Odds Ratio, adjusted Odds Ratio
ORIF: Open Reduction with Internal Fixation
RCT: Randomized Controlled Trial
SPDR: Swedish Prescribed Drug Register
SSI: Surgical Site Infection
